# Commencement of Montreal Veterinary College

**Published:** 1886-07

**Authors:** 


### COMMENCEMENT OF MONTREAL VETERINARY COLLEGE.

The final examinations, before a board of examiners appointed by the council of agriculture, took place in the college lecture room Tuesday morning. The board was constituted as follows: Messrs. Wm. Bryden, V.S., Boston, Mass.; Frank S. Billings, M.V., Roxbury, Mass.; Archibald McCormic, V.S., Ormstown; Charles C. Cowie, M.R.C.V.S., Ogdensburg, N. Y.; J. A. Couture,

V. S. Quebec; A. W. Harris, V.S., Ottawa; A. E. Cross, V.S., Montreal; Charles Levesque, V.S.. Berthier (en haut). The following gentlemen having passed all the examinations and attended the prescribed number of sessions, were considered by the board to be fully qualified to practice the science and art of veterinary medicine and surgery, and were judged worthy to receive the diploma of the college: Messrs. Wroughton, Whyte, Dyer, and Sangster.

The presentation of prizes and diplomas took place in the afternoon, Sir William Dawson, the principal of McGill university presiding. There was a large attendance of the friends of the college and students. Dr. Leclerc, secretary of the council of agriculture, read the report of the result of the various examinations and prizes. The chairman then presented the prizes to the successful competitors, and presented the diploma of the college to Messrs. T. A. Wroughton, C. C. Dyer, J. D. Whyte, and G. W. Sangster.

Sir William Dawson said though the number receiving diplomas was small, those who had received them might be congratulated on having gone through a most thorough course, for which they were greatly indebted to their instructors. It was a most fallacious method to judge a school by the number of its graduates. He hoped the time would come when, through Government aid, this school would cease to be a burden on its principal.

Mr. Blackwood, representing the Quebec Council of Agriculture, said that during the year he had had occasion to examine into the condition of the men who had been educated at this college, and he was gratified to learn that every one whom he had been able to trace had been successful. This was a remarkable record and an encouraging outlook to those now leaving the college, to whom as an old man he might be permitted to give one or two bits of advice. Certain temptations peculiarly surrounded the members of their profession. Let it be said of each of them, as he had once heard it remarked of their principal, that no money could affect his opinion of a horse; and let them be careful about drink, which it would be hard for them to avoid. He had himself in 1836 come to the conclusion that his only safe course lay in total abstinence, which course he had never departed from.

Dr. Billings, one of the examiners, had one thing to add to what had been said, and that was, if there was anything worse than rum it was the horse man. The veterinary surgeon who had his office in a stable and who spent his spare time in talking horse slang and soiling the floor with tobacco, disgraced his profession. The man who courted his clients in this way got the worst of it, while he who took the place where he belonged, at a respectable distance from them, would be respected by them. Dr. Billings ascribed to Principal McEachran the very first place on this continent as a veterinary surgeon and veterinary educator, and denounced the diploma mills and cheap subscription societies that passed for veterinary colleges all over the continent. He hoped to give his friend a race for the first place in the school about to be established by the State of Nebraska, He urged upon all students and graduates to devote themselves to original investigation. It was original investigation that gavè to Virchow the proud distinction which he enjoyed.

Dr. Hingston, in adding his quota of advice to the young men, said it was their duty to take the position of gentlemen among society and to uphold the dignity of their profession by their demeanor as well as by their acquirements. He quoted some eminent examples of veterinary men who took the highest social places.

Dr. McEachran endorsed the advice that had been given them, as well as what had been said of the success of the graduates. A number of these now held teaching positions, and although from his experience he could not recommend this occupation as a money-making one, they could at all events assure themselves that those who had passed through the course prescribed by this college were qnalified to teach.

The proceedings were then brought to a close.

Tne Montreal Veterinary Medical Association afterwards held a meeting for the purpose of conferring on the successful graduates the diploma of the association. In presenting them, the president, Dr. Baker, warmly congratulated the young men, saying he hoped they would always consider it an honor to possess these diplomas, and that the association would always be equally proud of the honors.

Mr. Feron was then elected to fill the offices of secretary-treasurer and librarian during the summer, after which the meeting adjourned.

